# A review of research on salivary biomarkers for oral cancer detection

**DOI:** 10.1186/2001-1326-3-3

**Published:** 2014-02-24

**Authors:** Yi-Shing Lisa Cheng, Terry Rees, John Wright

**Affiliations:** 1 Department of Diagnostic Sciences, Texas A&M University-Baylor College of Dentistry, 3302 Gaston Ave., Dallas, TX 75246, USA; 2 Department of Periodontics, Texas A&M University-Baylor College of Dentistry, 3302 Gaston Ave., Dallas, TX 75246, USA

**Keywords:** Saliva, Biomarker, Oral Cancer, Oral squamous cell carcinoma

## Abstract

Using saliva for disease diagnostics and health surveillance is a promising approach as collecting saliva is relatively easy and non-invasive. Over the past two decades, using salivary biomarkers specifically for early cancer detection has attracted much research interest, especially for cancers occurring in the oral cavity and oropharynx, for which the five-year survival rate (62%) is still one of the lowest among all major human cancers. More than 90% of oral cancers are oral squamous cell carcinoma (OSCC) and the standard method for detection is through a comprehensive clinical examination by oral healthcare professionals. Despite the fact that the oral cavity is easily accessible, most OSCCs are not diagnosed until an advanced stage, which is believed to be the major reason for the low survival rate, and points to the urgent need for clinical diagnostic aids for early detection of OSCC. Thus, much research effort has been dedicated to investigating potential salivary biomarkers for OSCC, and more than 100 such biomarkers have been reported in the literature. However, some important issues and challenges have emerged that require solutions and further research in order to find reliable OSCC salivary biomarkers for clinical use. This review article provides an up-to-date list of potential OSCC salivary biomarkers reported as of the fall of 2013, and discusses those emerging issues. By raising the awareness of these issues on the part of both researchers and clinicians, it is hoped that reliable, specific and sensitive salivary biomarkers may be found soon—and not only biomarkers for early OSCC detection but also for detecting other types of cancers or even for monitoring non-cancerous disease activity.

## Introduction

Worldwide, cancers of the oral cavity and pharynx are the 6^th^ most common type [1]. Although the incidence of oral and pharyngeal cancers is not considered high in the US (it accounts for approximately 3% of all malignancies in men and 2% of those in women) [1]-- its five-year survival rate has remained around 62%-- in contrast to the five-year survival rates for breast cancer (89%) and prostate cancer (99%) [2]. More than 90% of the oral cancers are squamous cell carcinomas (OSCC), which arise from the epithelial lining of the oral cavity. Even though the oral cavity is easily accessible for direct visual examination, most OSCCs are not diagnosed until an advanced stage, which is believed to be the major reason for the low survival rate. This underscores the importance of early and accurate detection by clinicians, as well as the need to educate the public about oral cancer risk factors and prevention.

In responding to the call for early detection of OSCC, several diagnostic adjuncts have been developed, or currently are in development, including the use of salivary biomarkers [3-5]. Saliva has been found to contain constituents that reflect the diseased or physiological state of the human body, and hence could be utilized for diagnostic purposes [6-8]. The search for reliable salivary biomarkers for early detection of OSCC has developed rapidly, spurred on by the fact that collecting saliva is relatively easy and non-invasive, compared to the drawing of blood. From the late 1990s until the present, more than 40 research studies have been published and more than 100 different salivary constituents have been suggested as potential OSCC salivary biomarkers (see Table 1). Several excellent reviews have been previously published concerning the basis for salivary diagnostics, and the history and evolution of salivary proteomic, transcriptomic, genomic, and metabolomic research for oral cancer detection [6,7,9-14]. This review article will provide an up-to-date list of potential OSCC salivary biomarkers, as of the fall of 2013, and will focus on some emerging issues/challenges that seem to be major roadblocks on the way to bringing reliable salivary biomarkers into actual clinical use. The intention of this review is to increase awareness of these issues on the part of both researchers and clinicians, with the hope that good solutions may be generated more quickly by group efforts in this field. Addressing these issues not only will serve to accelerate progress toward finding reliable salivary biomarkers for early OSCC detection, but also should have a positive effect on the search of reliable salivary biomarkers for other types of cancer detection or even for monitoring non-cancerous disease activity.

**Table 1:** Potential salivary biomarkers for oral cancer detection, reported as of 2013

**Category**	**Potential OSCC salivary biomarkers**	**Authors/year**
Non-organic compound	Na, Ca, F, and Mg	Shpitzer et al./2007 [55]
Peptide	Defensin-1	Mizukawa et al./1998 [28]
Proteins	P53 autoantibody	Warnakulasuriya et al./2000 [19]
	α-amylase	Chen et al./2002 [20]
	IL-8	St. John et al./2004 [23]
		Rhodus et al./2005 [24]
		Arellano-Garcia et al./2008 [70]
		Brinkmann et al./2011 [43]
		Elashoff et al./2012 [44]
	TNF-α	Rhodus et al./2005 [24]
	IL-1	
	IL-6	Rhodus et al./2005 [24]
		Katakura et al./2007 [34]
		Saheb-Jamee et al./2008 [35]
		Sato et al./2010 [36]
		Cheng et al./2013 [38]
	Basic fibroblast growth factor	Vucicevic et al./2005 [27]
		Gorugantula et al./2012 [39]
	Statherin	Contucci et al./2005 [62]
	Cyfra 21.1	Nagler et al./2006 [25]
	Tissue polypeptide antigen (TPA)	
	Cancer antigen 125 (CA125)	Nagler et al./2006 [25]
		Balan et al./2012 [57]
	Endothelin-1	Pickering et al./2007 [21]
		Cheng et al./2011 [40]
	IL-1β	Katakura et al./2007 [34]
		Brinkmann et al./2011 [43]
		Elashoff et al./2012 [44]
	CD44	Franzmann et al./2007 [46]
	Total salivary protein	Shpitzer et al./2007 [55]
	Insulin growth factor 1 (IGF-1)	
	MMP-2	
	MMP-9	Shpitzer et al./2007, 2009 [49,55]
	CD59	Hu et al./2008 [29]
	Catalase	
	Profilin	
	S100A9/MRP14	
	M2BP	Hu et al./2008 [29]
		Brinkmann et al./2011 [43]
		Elashoff et al./2012 [44]
	Carcinoembryonic antigen (CEA)	He et al./2009 [54]
	Carcinoma associated antigen CA-50	
	Salivary carbonyls	Shipitzer et al./2009 [49]
	Cyclin D1	
	Maspin	
	8-oxoguanine DNA glycosylase (OGG1)	
	Phosphorylated-Src	
	Ki-67	
	Lactate dehydrogenase	Shipitzer et al./2009 [49]
		Shetty et al./2012 [58]
	Transferrin	Jou et al./2010 [45]
	Zinc finger protein 501 peptide	Jou et al./2011 [60]
	Hemopexin	Jessie et al./2013 [59]
	Haptoglobin	
	Complement C3	
	Transthyretin	
	α1-antitrypsin	
DNAs	P53 gene codon 63	Liao et al./2000 [18]
	Loss of heterozygosity in the combination of markers D3S1234, D9S156, and D17S799	El-Naggar et al./2001 [51]
	Mitochondrial DNAs (cytochrome c oxidase I and cytochrome c oxidase II)	Jiang et al./2005 [37]
	Hypermethylation of promoters in tumor suppressor genes: DAPK, DCC, MINT-31, TIMP-31, TIMP-3, p16, MGMT, CCNA1	Carvalho et al./2011 [61]
mRNAs	IL-8	Li et al./2004 [22]
		Brinkmann et al./2011 [43]
		Elashoff et al./2012 [44]
	IL-1β	Li et al./2004 [22]
		Elashoff et al./2012 [44]
	DUSP1 (dual specificity phosphatase 1)	Li et al./2004 [22]
		Elashoff et al./2012 [44]
		Cheng et al./2013 [41]
	H3F3A (H3 histone family 3A)	Li et al./2004 [22]
		Elashoff et al./2012 [44]
	OAZ1 (ornithin decarboxylase antizyme 1)	Li et al./2004 [22]
		Elashoff et al./2012 [44]
		Cheng et al./2013 [41]
	S100P (S100 calcium binding protein P)	Li et al./2004 [22]
		Brinkmann et al./2011 [43]
		Elashoff et al./2012 [44]
		Cheng et al./2013 [41]
	SAT (spermidine/spermine N1-acetyltransferase EST)	Li et al./2004 [22]
		Brinkmann et al./2011 [43]
		Elashoff et al./2012 [44]
MicroRNAs	miR-125a	Park et al./2009 [31]
	miR-200a	
	miR-31	Liu et al./2012 [33]
Long non-coding RNAs	HOTAIR	Tang et al./2013 [56]
Oxidative stress-related molecules	Reactive nitrogen species (RNS) such as nitric oxide (NO), nitrites (NO_2_) and nitrates (NO_3_)	Bahar et al./2007 [26]
	Peroxidase	Bahar et al./2007 [26]
	Glutathione S-transferase (GST)	Bahar et al./2007 [26]
	Superoxide dismutase (SOD)	Agha-Hosseini et al./2012 [52]
	8-hydroxy-2-deoxyguanosine (8-OHdG)	
	Glutathione	Almadori et al./2007 [30]
	Malondialdehyde (MDA)	Agha-Hosseini et al./2012 [52]
Glucocorticoid	Cortisol	Bernabé et al./2012 [53]
Metabolomics	Cadaverine, alpha-aminobutyric acid, alanine, C5H14N5, piperidine, taurine piperideine, pipercolic acid, C4H9N, C8H9N, pyrroline hydroxycarboxylic acid, betaine, C6H6N2O2, leucine+isoleucine, tyrosine, histidine, tryptophan, beta-alanine, glutamic acid, threonine, serine, glutamine, choline, carnitine, C4H5N2O11P	Sugimoto et al./2010 [47]
	Phenylalanine	Wei et al./2011 [48]
		Sugimoto et al./2010 [47]
	Valine	Wei et al./2011 [48]
		Sugimoto et al./2010 [47]
	Lactic acid	Wei et al./2011 [48]
Glycosylation- related molecules	Sialic acid	Vajaria et al./2013 [32]
	α-L-fucosidase	
Other	Telomerase activity	Zhong et al./2005 [50]

## Review

Whole saliva (oral fluid) is unique and complex, both in its sources and composition. It consists not only of secretions from the three major salivary glands (parotid, submandibular and sublingual) and the minor glands, but also gingival crevicular fluid, oral mucosa transudate, secretions from nasal and pharyngeal mucosa, non-adherent bacteria, desquamated oral epithelial cells, keratin debris, blood cells, and perhaps food or medication residuals [8]. The functions of saliva include lubrication, digestion, antimicrobial activity, facilitating remineralization of the tooth enamel, and maintaining normal taste sensation [15]. These important functions are achieved by the various chemical components of saliva including water, inorganic compounds (ions), organic compounds (non-proteins and lipids), protein/polypeptides, and hormones [15]. Salivary proteins and polypeptides constitute a significant portion of the mix, and play an important role in carrying on the main functions of saliva. So far, more than 2300 proteins and peptides have been found in human saliva [16]. The most abundant proteins are α-amylase, albumin, cystatins, hystatins, secretory-IgA, lactoferrin, mucins, lysozymes, proline rich proteins, statherin and transferrin--which together account for more than 98% of the total salivary proteins [15,17]. Most of the potential OSCC salivary biomarkers are also salivary proteins (see Table 1). However, except for three, α-amylase, statherin, and transferrin, those proteins, as well as the non-protein OSCC salivary biomarker candidates, are present in a very low concentration in saliva and require methods/instruments with high sensitivity for detection.

### Reported potential salivary biomarkers for oral cancer detection

So far, more than 100 potential OSCC salivary biomarkers have been reported in the literature, based mainly on comparing the levels found in OSCC patients to the levels found in non-OSCC normal controls (see Table 1) [18-62]. Some of these potential OSCC salivary biomarkers have been investigated by more than one research group; however, in some studies, no significant differences have been found in IL-6 [23], IL-8 [34,35] and endothelin-1 [63] levels between the OSCC patients and their respective control groups.

The research methodology involved so far in investigating these potential OSCC salivary biomarkers can be grouped according to the types of biomarker, as follows:

1. *Non-organic compound biomarkers*

Flame photometry, atomic absorption, and spectrophotometry [55]

2. *Peptide or protein biomarkers*

High performance liquid chromatography (HPLC) [28,62]

Enzyme-linked immunosorbent assay (ELISA) [19,21,23,24,27,34-36,39,40,46,49,54,55,57]

Radio-immunoassay [25]

Two-dimensional gel electrophoresis (2DE), followed by mass spectrometry (MS) [59]

2DE and reverse-phase liquid chromatography (LC), followed by LC-tandem MS [29]

Matrix-assisted laser desorption/ionization time-of-flight mass spectrometry (MALDI-TOF MS) [20,60]

2DE followed by MALDI-TOF MS [45]

3. *DNA, mRNA or microRNA biomarkers*

Polymerase chain reaction (PCR) [18,37,51]

Quantitative PCR (qPCR) [31,33,41-44,56]

Microarrays followed by qPCR [22]

4. *Metabolomic biomarkers*

Capillary electrophoresis TOF MS [47]

HPLC with quadrupole/TOF MS [48]

5. *Miscellaneous biomarkers (chemical and enzyme activity)*

HPLC [30]

Colorimetric (mostly commercially available) assays [26,32,49,50,52,53,58]

Most OSCC salivary biomarker research has involved investigating the constituents of the whole saliva in an unstimulated state, although two studies did investigate the stimulated saliva samples [28,36]. After a saliva sample is collected, a centrifugation processing procedure is often performed to remove the solid constituents (desquamated oral epithelial cells, keratin debris, blood cells, bacteria and food residuals, if any), but some studies appear to have analyzed the whole saliva content without centrifugation [46,54,58,63,64]. After separating out those solid constituents, samples were often stored in a frozen state until further analysis. Most salivary biomarker research studies have investigated only the supernatant (cell-free) portion of the saliva samples, while other studies investigated only the pellet portion of the saliva [50,51,61] or both the supernatant and the pellet portions [49] after centrifugation.

### Issues/challenges in OSCC salivary biomarker research

#### 1. A lack of standardization of conditions and methods of saliva sample collection, processing, and storage

Despite the fact that more than 100 potential OSCC salivary biomarkers have been reported, there has been no standardization regarding the condition of the subjects from whom the saliva samples are collected (e.g., the timing in regard to prior food and drink intake, or the use of oral hygiene products). Similarly, a uniform method has not been established for how the saliva samples are collected, processed and stored prior to measurement and comparison of the biomarker levels in the groups studied.

For example, in regard to participant requirements at the time of saliva collection, all researchers collected saliva samples in the morning, yet some researchers collected saliva samples from participants at least one hour after food intake [23,25,26,63]; some collected saliva at least 1 1/2 hours after food intake [24]; and in ours and another study, we collected saliva in the morning before the participants ate, drank or performed any oral hygiene procedures [30,39-41]. In regard to saliva processing methods, most investigators centrifuged the saliva samples immediately after collection. However, they carried out this procedure with various degrees of centrifugal force and for various lengths of time, such as 800 g for 10 minutes [25,26,49]; 2000 g for 10 minutes [52]; 2600 g for 15 minutes [23,32,40,41,43]; 14,000 g for 20 minutes [59]; 2000 rpm (gravity not specified) for 5 minutes [53]; 3500 rpm (gravity not specified) for 15 minutes [34]; 14,000 rpm (gravity not specified) for 20 minutes [21]. Others stored the whole saliva samples at −80°C immediately after collection, then later thawed and centrifuged the samples at 4500 g for 20 minutes [35] or 6000 rpm (gravity not specified) for 20 minutes [24]. In regard to saliva sample preservation, we and some other investigators added proteinase inhibitors or RNase inhibitors to preserve salivary proteins and RNAs, respectively [22,29,39-41], but most investigators did not add any inhibitors. In regard to saliva sample storage, most researchers stored the saliva samples in −80°C, although others stored samples at −20°C [28,52,54] or 4°C [30].

The differences in these factors among the different studies raises the question as to whether the levels of the potential OSCC salivary biomarkers reported in any one lab could be compared to the levels of the same biomarker reported in any other lab. In fact, for the reported potential biomarkers which have been investigated by more than one study, a wide variability was found in the levels of both the diseased and the control groups among different labs, and the different methods used for collecting and handling the saliva samples are likely to be one of the reasons for that variability. As research efforts will continue to be devoted to this promising field, standardization of saliva collection, processing and storage methods is essential, so that all the research findings among different research groups can be compared and validated, and progress accelerated toward finding the most reliable validated OSCC salivary biomarkers. Without such standardization and validation of biomarkers, valuable research resources are being squandered, because reported results cannot be translated into the desired clinical use and patient outcomes. A panel of experts in this research field is needed to discuss this issue and find a way to establish the standards needed.

#### 2. Variability in the levels of potential OSCC salivary biomarkers in both non-cancerous individuals and OSCC patients, suggest unknown confounding factors

The wide variations noted in the reported levels of some of the salivary biomarker candidates among different research labs obviously creates difficulties in determining the reference level and the OSCC level for any given potential OSCC salivary biomarker. For example, salivary IL-6 and IL-8 are two of the most studied biomarkers, not only for potential OSCC detection but also for monitoring disease activity of chronic periodontitis and oral lichen planus (OLP)--a chronic mucocutaneous inflammatory disease that affects about 2% of the world population [65,66]. As can be seen in Table 2[23,24,34,35,38,67-72], wide variations in the reported salivary IL-6 and IL-8 levels among the different studies were noted even in the healthy controls, with the average IL-6 levels ranging from 1.4 ± 0.9 pg/ml [24] to 47.46 ± 18.74 pg/ml [67], and IL-8 levels ranging from 250 pg/ml [23,34,35] to 1945 ± 181 pg/ml [68]. When the reference levels vary so much among the different studies, it is impossible to determine what ranges of salivary IL-6 or IL-8 levels are likely to indicate OSCC development. For example, the average salivary IL-8 levels found in OSCC patients in two studies were 720 pg/ml [23] and 1093.7 ± 1089.0 pg/ml [35], respectively --in contrast to 250 pg/ml and 700.7 ± 1031.5 pg/ml, respectively, in healthy controls. The average salivary IL-8 levels found in chronic periodontitis and healthy controls reported by Teles et al.[68] were 2268 ± 111 and 1945 ±181 pg/ml, respectively, both of which would most likely be considered in the concentration range for OSCC by the two previous studies reported by St John et al. [23] and SahebJamee et al. [35].

**Table 2:** Salivary Concentrations of IL-6 and IL-8 (pg/ml) in OSCC, OLP, and chronic periodontitis patient groups

**Author/reographical region/year**	**OSCC**		**OLP**		**Chronic periodontitis**		**Controls**	
	**IL-6**	**IL-8**	**IL-6**	**IL-8**	**IL-6**	**IL-8**	**IL-6**	**IL-8**
**St. John et al./California, USA/2004**[23]		720						250
**Rhodus et al./Minnesota, USA/2005**[24]	88.2± 43.2	3154.1±1023.2					1.4±0.9	1580± 789
**Rhodus et al./Minnesota, USA/2005**[67]			371.35±205.52	2194.3± 496.7			47.46±18.74	703.8± 131.6
**Katakura et al./Tokyo, Japan/2007**[34]	86.5	720					0	250
**Zhang et al./Sichuan, China/2008**[69]			48.79±8.53	1737.49±1073.54			29.9±4.68	641.46±172.91
**Saheb Jamee et al./Tehran, Iran/2008**[35]	40.9± 79.5	1093.7±1089.0					2.5±1.3	700.7± 1031.5
**Arellano- Garcia et al./California, USA/2008**[70]		3347.7±2929						759.4± 563
**Teles et al./Massachusetts, USA/2009**[68]						2268±111		1945±181
**Sharma et al./Manipal, India/2011**[71]					311.35±11.51		17.15±8.44	
**Ebersole et al./Kentucky, USA/2013**[72]					35.57±48.17		3.30±2.32	
**Cheng et al./Texas, USA/2014**[38]	178.41±172.32	1525.33±1123.95	20.74±22.28	1328.37±731.80	5.85±4.02	738.79±394.00	4.92±8.77	890.83±563.22

All studies investigated unstimulated whole saliva.

These wide variations in the levels of the same salivary constituent across the different research studies could be due at least partly to the different processing methods used, as described above. However, they may also possibly be due to inherent biological variations within different individuals and groups. In fact, some studies have reported intra- and inter-subject variability in salivary proteome and in specific potential OSCC salivary protein and mRNA biomarkers in studies of healthy adults [73-75]. Inherent biological variations are known to create difficulties in determining the reference values for biological samples in clinical laboratories [76]. Such variations in salivary constituents could be attributed to differences in ethnic background, geographic locations, age, gender, non-neoplastic systemic diseases, dietary habits, medications being taken or other factors. For example, significantly increased levels in one of the potential salivary OSCC salivary biomarkers, endothein-1, have been reported in patients with chronic heart failure [77] or upper gastrointestinal diseases such as gastric ulcer, duodenal ulcer and gastritis [78] in the absence of OSCC. Age-related reduction in the concentrations of certain salivary proteins [79,80] has also been reported, although whether there are any significant age-related changes in any of the reported potential OSCC salivary biomarkers has not been determined. In summary, the intra- and inter-subject variability points to an urgent need for more research regarding possibly underlying causes for these inherent biological variations, in order to refine and determine the appropriate reference range for any potential OSCC salivary biomarkers.

#### 3. The need for further validation of OSCC salivary biomarkers

##### *Validation in the presence of common oral inflammatory conditions*

Ideally, a good clinical test requires high sensitivity and specificity. The oral cavity is commonly subject to inflammation from a variety of causes, including trauma, dental plaque, infection and certain mucocutaneous inflammatory diseases. Whether such oral inflammation (non-neoplastic conditions) affects the levels of the potential OSCC salivary biomarkers is essentially unknown, because most studies have investigated the potential salivary biomarker levels only in OSCC patients and non-OSCC controls, without regard for other inflammatory conditions that might have been present [9,21-27]. If the levels of any of the potential OSCC salivary biomarkers are increased in the presence of oral inflammation to a degree that there is no significant difference between those levels and the levels found in OSCC patients, it would result in a high false positive rate and greatly reduce the value of that biomarker in clinical use for OSCC detection.

In review of the reported potential OSCC salivary biomarkers, many of them-- such as *IL-6*[24,34,35], *IL-8*[23,24,70], *IL-1β*[22,70], *basic fibroblast growth factor*[27], and *molecules related to oxidative stress*--[26,30] are known to be important factors involved in inflammation and/or wound healing [81,82]. Indeed, the levels of some of these salivary constituents have been reported to be significantly higher or lower in periodontitis or OLP patients who did not have OSCC [67,69,71,72,83,84]. Therefore, research that validates any potential OSCC salivary biomarker with individuals having common non-neoplastic oral inflammatory diseases is necessary in order to establish the reliability of that salivary OSCC biomarker.

##### *Validation in the presence of other types of human cancers*

The advantages (being non-invasive and easy to collect) of using saliva over blood for disease detection and monitoring of disease progression encourage salivary biomarker research activities not only for OSCC detection but also for detection of other types of cancers. For instance, several potential salivary biomarkers for breast and lung cancer detection have been reported in recent years [85-92]. Some of the reported salivary biomarkers for breast carcinomas, such as CA125 [91], haptoglobin, profilin-1 and transferrin [87]; and one reported salivary biomarker for lung cancer, S100 calcium binding protein A9 [86], are the same ones reported as potential OSCC salivary biomarkers [25,29,57,59]. Salivary annexin A1, carbonic anhydrase VI, S100 calcium binding protein, and lipocalin have also been reported as potential biomarkers for both breast and lung cancers [86,87]. These findings suggest that the levels of at least some salivary constituents could be significantly changed by the presence of more than one type of cancer, a fact which points to the necessity of validating the specificity of the reported potential OSCC salivary biomarkers with patients who have other types of cancers.

## Conclusion

Salivary biomarkers represent a promising non-invasive approach for oral cancer detection, and an area of strong research interest. However, some issues/challenges have arisen that need to be resolved in order to establish this approach as a reliable, highly sensitive and specific method for clinical use. These issues include a lack of standardization for saliva sample collection, processing, and storage; wide variability in the levels of potential OSCC salivary biomarkers in both non-cancerous individuals and OSCC patients; and a need for further validation of OSCC salivary biomarkers with individuals who have either a chronic oral inflammatory disease or other types of cancers, but do not have OSCC. These issues call for convening a panel of researchers in this field to aim for eventual standardization, plus further research, especially concerning biological variance and physiological changes affecting the potential oral cancer salivary biomarkers. The experience gained in OSCC salivary biomarker research also can serve as an important reference in salivary diagnostics, including identifying, validating, and applying salivary biomarkers for other types of cancer detection and for monitoring non-cancerous disease activity.

## Abbreviations

OSCC: Oral squamous cell carcinoma; OLP: Oral lichen planus; HPLC: High performance liquid chromatography; ELISA: Enzyme-linked immunosorbent assay; RIA: Radioimmunoassay; MALDI-TOF MS: Matrix-assisted laser desorption/ionization time-of-flight mass spectrometry; 2DE: Two-dimensional gel electrophoresis; qPCR: Quantitative polymerase chain reaction.

## Competing interests

The authors declare that they have no commercial or other competing interests to disclose.

## Authors’ contributions

The author YSLC reviewed and analyzed the published research studies, conceptualized and drafted the manuscript. TR and JW contributed important intellectual content for the manuscript. All three authors have been involved in revising the manuscript. All authors read and approved the final manuscript.
